# Two Cases of Intraoperative ECG Artifact Mimicking Ventricular Tachycardia and How to Know When It Is Real

**DOI:** 10.7759/cureus.38773

**Published:** 2023-05-09

**Authors:** Alexander M DeLeon, Jessica J Weeks, Lydia Su, Vicente Garcia Tomas

**Affiliations:** 1 Anesthesiology, Northwestern Memorial Hospital, Chicago, USA; 2 Regional Anesthesiology, Northwestern Memorial Hospital, Chicago, USA

**Keywords:** cardiac defibrillators, arrhythmias, electrocardiogram, ventricular tachycardia, electrical artifact

## Abstract

Electrocardiographic (ECG) artifacts may resemble ventricular tachycardia (VT), leading to inappropriate therapies. Despite extensive training, electrophysiologists have still been shown to misinterpret artifacts. The literature is scant regarding the intraoperative identification by anesthesia providers of ECG artifacts resembling VT. We present two cases of the intraoperative occurrence of ECG artifacts resembling VT. The first case involved a patient undergoing extremity surgery after receiving a peripheral nerve block. The patient was treated with a lipid emulsion for a presumptive local anesthetic systemic toxicity diagnosis. The second case was a patient with an implantable cardiac defibrillator (ICD) with suspended anti-tachycardia functionality due to the location of the surgery in the region of the ICD generator. The second case's ECG was identified as an artifact, and no treatment was initiated. Misinterpretation of intraoperative ECG artifacts continues to lead clinicians to institute unnecessary therapies. Our first case occurred in the context of a peripheral nerve block leading to the misdiagnosis of local anesthetic toxicity. The second case occurred during the physical manipulation of the patient during liposuction.

## Introduction

Despite reports of electrocardiographic (ECG) artifacts being interpreted as ventricular tachycardia (VT), clinicians continue to misinterpret such artifacts occasionally leading to inappropriate, invasive therapies [1].

We present two cases of intraoperative ECG artifacts in patients at increased risk for VT without electrical equipment malfunctions. Each case is unique relative to the published literature, yet both circumstances occur with enough frequency to warrant this report. The first case followed a peripheral nerve block resulting in the emergent administration of lipid emulsion therapy per the ASA Practice Advisory for treating local anesthetic systemic toxicity [2]. The second case occurred in a patient with a history of documented cardiac arrest requiring an implantable cardiac defibrillator (ICD) that was disabled due to the location of the surgical field per the ASA Practice Advisory for ICD management [3]. The CARE Checklist was followed in the preparation of this manuscript.

## Case presentation

Case 1

A 61-year-old, 66 kg female patient presented for an open reduction internal fixation of a distal radius fracture. Her past medical history was significant hypertension and diabetes mellitus. An ultrasound-guided supraclavicular nerve block with 20 mL of 0.5% bupivacaine was performed preoperatively for anesthesia after pre-procedural sedation with midazolam. The patient's vital signs were stable throughout the peripheral nerve block procedure.

**Figure 1:**
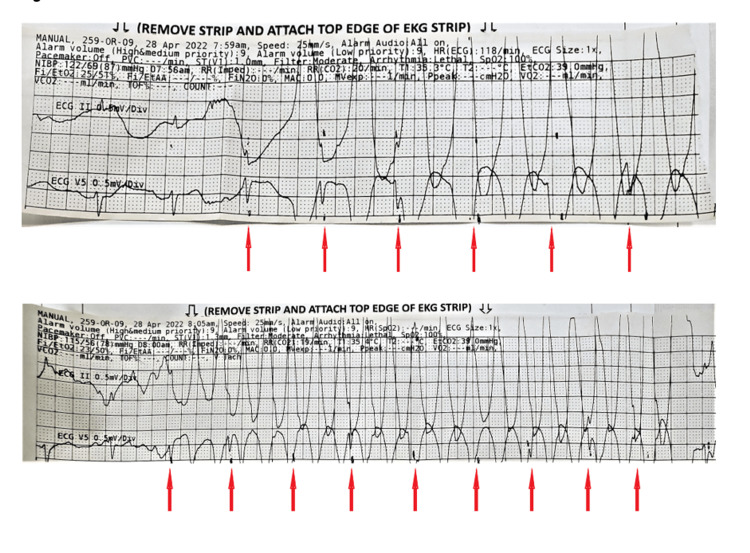
Rhythm strips Electrocardiogram strips printed intraoperatively show artifacts mimicking ventricular tachycardia in leads II and V. Regularly appearing notching (indicated by the red arrows) within the abnormal rhythm suggests the presence of artifacts.

Intraoperatively, one hour after the completion of the nerve block, the patient was noted to have multiple episodes of non-sustained ventricular tachycardia (Figure 1). Each episode lasted 10 to 15 beats. The surgical field was observed during these episodes, and no surgical manipulation was occurring. The patient remained hemodynamically stable. Given the context of a peripheral nerve block, the suspicion of local anesthetic system toxicity (LAST) indicated the administration of lipid emulsion. A 20% lipid emulsion bolus of 100 mL was administered over three minutes, followed by an infusion of 15 mL/min for a total volume of 500 mL. After administration of lipid emulsion, the VT episodes resolved. The surgeons were notified about the concern for VT. The critical part of the surgical procedure was nearly completed at that point, and the patient remained hemodynamically stable through the short runs of suspected VT, so the decision was made to expedite the remainder and close. The procedure concluded uneventfully.

The intraoperative electrocardiographic strips were reviewed with a cardiologist, revealing subtle QRS complexes buried within what was categorized as an electrocardiographic artifact (Figure 1). Postoperative laboratory tests, including troponin and brain natriuretic peptide, were unremarkable.

The patient demonstrated no sequelae of receiving lipid emulsion and was discharged uneventfully. On follow-up with her orthopedic surgeon, she showed no long-term adverse effects.

Case 2

A 64-year-old woman presented for cosmetic bilateral breast reduction and liposuction therapy. Her medical history was significant for well-controlled hypertension, stable pulmonary sarcoidosis, and a history of cardiac arrest at home four years prior. She received cardiopulmonary resuscitation from her husband regarding the patient's cardiac arrest. Upon the emergency medical service's arrival, ventricular fibrillation was identified, and the patient received four therapeutic rounds of external cardiac defibrillation. The patient remained comatose for five days, followed by awakening and experiencing a full neurologic recovery. Upon workup, the patient was found by cardiac catheterization to have no significant stenotic lesions. Her initial transthoracic echocardiogram demonstrated a severely depressed ejection fraction.

The patient received a Boston Scientific bi-ventricular automated intracardiac defibrillator (AICD, G158 Dynagen X4 CRT-D; Boston Scientific, Marlborough, Massachusetts). One year after the initial cardiac event, her ejection fraction recovered to 52%, and she continued to show no evidence of cardiac sarcoidosis.

All vital signs were within normal limits. Her medications were significant for carvedilol and losartan. Based on the location of the ICD generator and the surgical field, including the entire anterior chest area, the decision was made, in consultation with the electrophysiology service (EP), to suspend the anti-tachycardia functions of the ICD. The EP service changed the ICD settings in the operating room once external pacemaker pads and five lead ECG monitoring were assured. The pacemaker functions were active, given that the patient received resynchronization therapy from her device and had an underlying rhythm of 60 bpm. External defibrillator pads were placed along the mid-axillary lines on each of the patient's sides due to restrictions related to the sterile field. The external defibrillator unit was immediately available.

**Figure 2:**
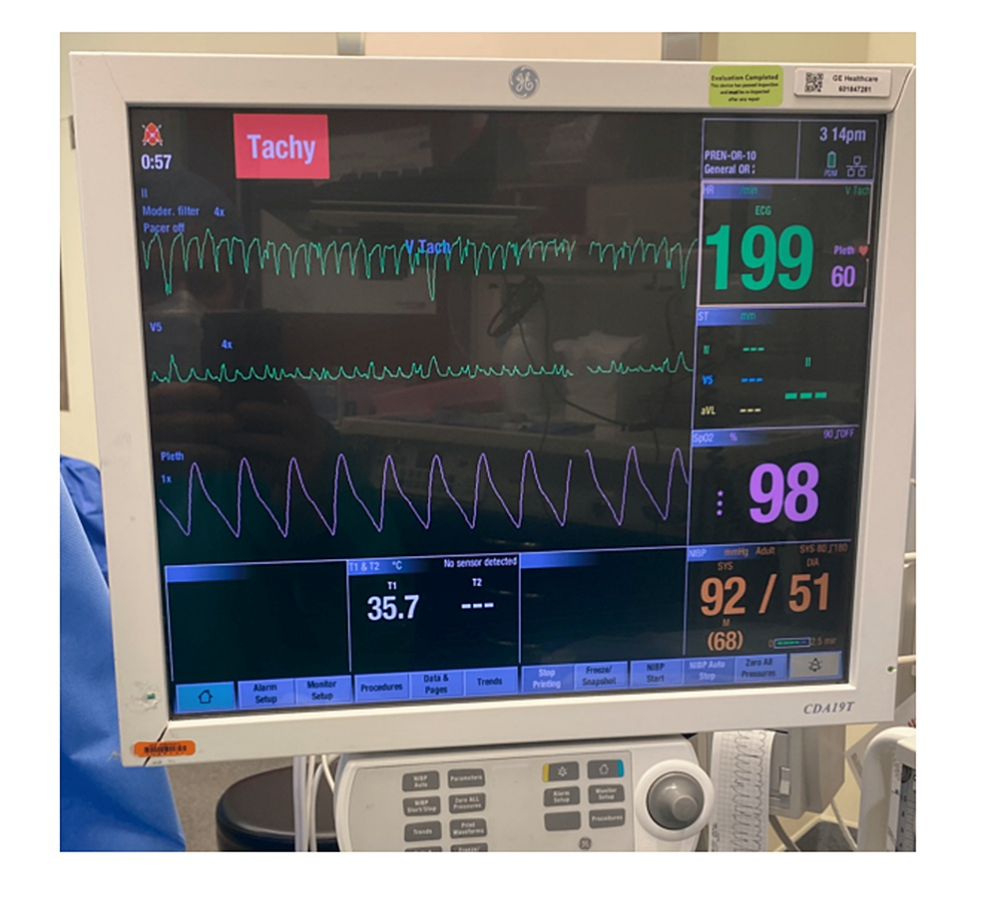
Intraoperative anesthesia monitor The two visible ECG leads demonstrate a wide-complex tachycardia pattern. The monitor's algorithm interpreted the ECG as V-tach. The pulse oximetry waveform and blood pressure are normal during the event, indicating this to be an artifact. Also, the surgeons performing the repetitive motion of liposuction led to the diagnosis of artifact vs. actual VT.

One hour and thirty minutes after the surgical incision, the intraoperative ECG monitor alarmed "V-tach" and displayed a tracing resembling VT (Figure 2). The BP was 92/51 mmHg, and the HR based on the pulse oximeter was 60 bpm despite the ECG reading of 199 bpm heart rate. At the time of the event, the intraoperative anesthesia provider observed the surgeon performing a repetitive movement (liposuction) in the area of the ICD generator. The event was interpreted as an artifact based on the stable hemodynamics, continuous pulse observed through the pulse oximeter waveform, and the surgical manipulation coinciding. No external defibrillation was indicated nor performed.

Upon completion of the surgical procedure, the anti-tachycardia functions were restored in the operating room. The surgical procedure was completed without incident, and the patient was discharged to her postoperative room per hospital routine.

## Discussion

The presented cases illustrate that ECG artifacts may mimic malignant arrhythmias and are a clinical challenge facing anesthesia providers. Anesthesia providers are at particular risk of mistreating an artifact because they continually monitor and interpret intraoperative ECG tracings that can often be influenced by patient movement and electrical artifacts. Also, anesthesia providers frequently initiate therapies, such as peripheral nerve blocks, with associated sequelae that may present as ventricular tachyarrhythmias. Radley et al. reported the consequences of 12 misinterpretations of the artifact as VT [1]. All 12 reported cases underwent unnecessary diagnostic or therapeutic interventions. None of the 12 cases occurred intraoperatively or involved anesthesia personnel. Three patients underwent cardiac catheterization. Separate patients each received an implantable cardio-defibrillator (ICD), a pacemaker, and a precordial thump. The remainder of the 12 patients received unnecessary medical therapies, including antiarrhythmic medications [1].

Knight et al. published a study investigating the incidence of misinterpretation of the artifact as VT [4]. In their study, internists, cardiologists, and electrophysiologists interpreted a standardized artifact ECG simulating a wide-complex tachycardia. Despite the electrophysiologists being the most accurate of the three groups, 38% still misinterpreted the ECG as ventricular tachycardia. Ninety-four percent of the responding internists incorrectly identified the ECG, and 58% of the general cardiologists misinterpreted the ECG. Such findings illustrate the challenges with interpreting ECG artifacts.

Regarding the published literature on intraoperative ECG artifacts, two cases of artifacts mimicking ventricular tachycardia were reported during endoscopic sinus surgeries [5,6]. In each case, the artifact occurred during the micro debridement of intranasal and sinus structures. ECG artifacts mimicking tachyarrhythmias have also been reported to be associated with somatosensory-evoked potentials monitoring, cardiopulmonary bypass, and high-frequency oscillatory ventilation [7-10]. A review of equipment-related ECG artifacts was published by Patel et al. [11]. The review discusses equipment movement, electrical current leakage, grounding failure, and interference by capacitance as potential causes for ECG artifacts. No case of liposuction-associated ECG artifact mimicking VT has been reported in the literature.

The primary limitation of the current report is the nature of case reports. These cases represent two intraoperative cases of artifacts mimicking VT, yet given the dynamic and multivariate nature of the operating room setting; misinterpretation may still occur.

## Conclusions

We report two unique intraoperative cases of VT artifacts leading to differing outcomes. The first case is notable because it is the first report of lipid emulsion therapy given in response to VT artifact for treating presumed LAST. The second case is noteworthy because it is the first report of liposuction-induced VT artifact. Differentiating ECG artifacts from actual VT should focus on the hemodynamics, including the pulse oximetry waveform and blood pressure. The surgical field should also be observed for the relation between movement and the occurrence of the ECG abnormality.
